# Effect of polycystic ovary syndrome on the life quality of young women

**DOI:** 10.1590/1806-9282.20231368

**Published:** 2024-05-03

**Authors:** Özden Tandoğan, Eda Yakit Ak, Arzu Akdemir, Ümran Oskay, Nihal Callioglu

**Affiliations:** 1 Istanbul Arel University, Faculty of Health Sciences, Department of Nursing – İstanbul, Turkey.; 2 Dicle University, Atatürk Health Services Vocational School – Diyarbakır, Turkey.; 3 Uskudar University, Faculty of Health Sciences, Department of Midwifery – İstanbul, Turkey.; 4 Istanbul University-Cerrahpaşa, Florence Nightingale Faculty of Nursing, Department of Women's Health and Diseases Nursing – İstanbul, Turkey.; 5 University of Health Sciences, Basaksehir Cam and Sakura City Hospital, Department of Obstetrics and Gynecology – İstanbul, Turkey.

**Keywords:** Polycystic ovary syndrome, Quality of life, Lifestyle factors, Women

## Abstract

**OBJECTIVE:**

The study evaluated the opinions of polycystic ovary syndrome on the life quality of women.

**METHODS:**

A total of 249 women with polycystic ovary syndrome participated in this descriptive study between October 2022 and July 2023 in Istanbul, Turkey.

**FINDINGS:**

Polycystic Ovary Syndrome and Quality of Life was significantly correlated with age (p=0.000) and frequent weight loss diets (p=0.000) (p<0.01). Among the Polycystic Ovary Syndrome and Quality of Life total score and polycystic ovary syndrome symptoms, those with hormone imbalance and insulin resistance had the highest mean scores, while those with menstrual irregularity and fatigue had the lowest.

**CONCLUSION:**

Advancing age changes the quality of life of women with polycystic ovary syndrome. To prevent the negative impact of polycystic ovary syndrome on women's quality of life, it is recommended that health professionals develop effective care plans utilizing available evidence.

## INTRODUCTION

Polycystic ovary syndrome (PCOS) is a chronic endocrine and metabolic disorder common in women, consisting of polycystic changes in the ovaries, menstrual cycle disorder, insulin resistance, obesity, and hyperandrogenism^1^. Prevalence varies between 6 and 20% depending on the diagnostic criteria^2^.

Individual assessments of physical, psychological, and social well-being should be incorporated into the health-related quality of life concept. For example, women with hirsutism are reported to have higher rates of psychological morbidity, social fear, and anxiety than women in the control population, and being infertile has a negative impact on sexual functioning and mood^3^. Therefore, all psychological, social, and cultural needs of young women diagnosed with PCOS should be addressed in a holistic approach^4^.

Many studies have shown that PCOS symptoms reduce the quality of life overall as they are painful, uncomfortable, and culturally associated with traits defined as unfeminine and undesirable^5–7^. Despite this, it has been suggested that the negative impact of PCOS symptoms on quality of life has been largely overlooked^8^. Although PCOS has a negative impact on quality of life, this impact varies from culture to culture. In Turkish women with PCOS, irregular menstrual cycles and hirsutism had a major impact on quality of life, whereas, in Iran, menstrual irregularities and infertility were more effective in reducing quality of life^4,8^. For Brazilian women with PCOS, body weight and infertility were reported to have the greatest negative impact on quality of life^9^. In a study on Italian women, PCOS patients with obesity reported significantly deteriorating their quality of life^4^. The impact of PCOS on quality of life may be specific and vary in different cultures. International evidence-based guidelines recommend that health professionals assess and consider the perception of symptoms, impact on quality of life, and personal priorities for care to improve patient outcomes^6^.

Health professionals must provide care with a holistic approach that focuses on how the emotional, physical, and social problems experienced by women of different cultures, geographical origins, and traditions due to PCOS symptoms affect their daily lives. In addition, investigating women's perceptions about their treatment and life quality by age is necessary for delivering individualized healthcare services. This research may raise awareness among healthcare professionals and enable them to support women by considering that life quality varies depending on age. Therefore, this study aimed to determine the quality of life of women with PCOS.

## METHODS

This cross-sectional study was conducted between October 2022 and July 2023. The study population consisted of women diagnosed with PCOS aged 18 years and older living in Turkey. In the systematic review study of Deswal et al.^10^ the prevalence of PCOS in women of reproductive age was taken with the unknown sampling method, the sample number calculated with a 95% confidence level and 0.05 sensitivity was determined as 249, and the study was completed with 250 women^11^. Ethical and institutional approval for the study (2022.10.19) was obtained (E-71273842-903.07.05-514118). Descriptive Information Form and Polycystic Ovary Syndrome and Quality of Life-50 Scale (PCOSQ-50), prepared in line with the literature, were used as data collection tools. Research data were collected through an online form (Google form). Participants were invited to the study electronically. The women in this study self-reported having PCOS.

The descriptive information form consisted of 25 questions developed by the researchers to determine women's socio-demographics, general health status, and PCOS history^11^.

PCOSQ-50 was developed by Nasiri-Amiri et al.^12^. Turkish validation was conducted by Koyutürk^13^. Cronbach's alpha of the PCOSQ-50 scale was reported to be 0.972. In our study, Cronbach's alpha value was 0.926. The data were analyzed with SPSS 21.0 and 95% confidence level. The t-test and ANOVA test were used to analyze the differences in scale scores according to demographic characteristics.

## RESULTS

The mean age of the women who participated in the study was 28.47±6.21 years. Notably, 81.5% of the women were university graduates or above. Among the participants, 61.0% were employed, and the most common occupations were civil servants (25.3%) and students (20.5%), respectively.

Among the participants, 30.9% had another disease other than PCOS, and the mean number of years since diagnosis of PCOS was 7.30±5.79. Regarding the symptoms of PCOS, 61.4% of the participants reported hair growth, 72.3% reported menstrual irregularity, 44.2% reported obesity, 49.8% reported acne pimples, 54.2% reported hair loss, 48.6% reported insulin resistance, 52.6% reported hormone imbalance, and 71.1% reported fatigue.

The characteristics of the women who participated in our study and the comparison of PCOSQ total and sub-dimensions are presented in Table 1. For PCOSQ total, those with hormone imbalance and insulin resistance had the highest mean scores for PCOS symptoms, while those with menstrual irregularity and fatigue had the lowest (Table 2).

**Table 1:** Comparison of some characteristics of women and Polycystic Ovary Syndrome and Quality of Life scores (n=249).

		Psychosocial and Emotional		Fertility		Sexual Function		Obesity and Menstrual Disorders		Hair Growth		Coping		PCOSQ Total	
		X	SD	X	SD	X	SD	X	SD	X	SD	X	SD	X	SD
Age (years)	≤25	3.13	0.90	2.43	1.03	2.09	0.91	3.11	1.07	3.35	1.36	2.68	1.04	2.90	0.85
	26–30	3.15	0.83	2.76	1.11	2.52	1.03	3.38	1.04	3.44	1.28	2.81	1.11	3.03	0.83
	31–35	2.64	0.83	2.27	0.95	2.13	0.76	2.91	0.88	2.60	1.24	2.23	0.93	2.49	0.68
	≥36	2.52	0.78	1.88	0.77	2.27	0.89	2.88	0.91	2.34	1.27	1.97	0.75	2.35	0.63
	F	7.561		6.395		2.536		2.860		8.879		7.402		8.581	
	p-value	0.000*		0.000*		0.058		0.038*		0.000*		0.000*		0.000*	
Work	Working	2.85	0.88	2.33	0.97	2.18	0.90	3.04	1.01	2.94	1.35	2.42	1.02	2.67	0.80
	Not working	3.15	0.87	2.59	1.13	2.47	0.96	3.26	1.03	3.39	1.35	2.73	1.07	2.98	0.83
	t	-2.669		-1.864		-1.988		-1.656		-2.569		-2.319		-2.964	
	p-value	0.008*		0.064		0.048*		0.099		0.011*		0.021*		0.003*	
Currently not having any disease other than PCOS	Yes	3.11	0.87	2.51	1.05	2.46	0.87	3.36	0.99	3.17	1.31	2.64	1.07	2.92	0.79
	No	2.90	0.89	2.39	1.04	2.19	0.95	3.02	1.02	3.09	1.39	2.50	1.04	2.73	0.83
	t	1.742		0.783		1.831		2.432		0.424		1.010		1.696	
	p-value	0.083		0.435		0.069		0.016*		0.672		0.314		0.091	
Time to diagnosis of PCOS (years)	5 years and less	3.10	0.88	2.49	1.03	2.46	0.90	3.24	1.00	3.17	1.39	2.73	1.08	2.93	0.83
	6–10 years	3.09	0.74	2.54	0.99	2.15	0.98	3.18	0.98	3.42	1.27	2.61	0.93	2.88	0.70
	10 years	3.00	0.86	2.58	1.04	2.44	0.81	3.39	0.85	3.16	1.31	2.57	0.99	2.89	0.71
	F	0.303		0.159		1.929		0.702		0.802		0.556		0.078	
	p-value	0.739		0.853		0.149		0.497		0.45		0.574		0.925	
Family history of PCOS	Yes	3.00	0.94	2.57	1.14	2.26	0.89	3.26	1.01	3.54	1.45	2.54	1.14	2.90	0.87
	No	2.95	0.87	2.39	1.01	2.28	0.94	3.09	1.03	2.99	1.32	2.54	1.03	2.76	0.81
	t	0.366		1.105		-0.125		1.105		2.686		-0.004		1.112	
	p-value	0.715		0.27		0.901		0.27		0.008*		0.997		0.267	
Frequency of hospital visits for PCOS control	36 months	2.87	0.95	2.38	1.10	2.26	0.85	3.10	1.06	3.24	1.38	2.38	1.14	2.75	0.89
	1 year	2.88	0.91	2.23	0.94	2.08	0.85	3.01	1.04	2.96	1.34	2.46	1.03	2.68	0.83
	Depends on the situation	3.06	0.83	2.57	1.02	2.33	0.97	3.22	0.96	3.19	1.36	2.65	1.01	2.89	0.78
	F	1.302		2.165		0.950		0.898		0.619		1.423		1.330	
	p-value	0.274		0.117		0.389		0.409		0.539		0.243		0.267	

X: mean; SD: standard deviation; F: one-way ANOVA; t: Student's t.

* p<0.05.

**Table 2:** Investigation of Polycystic Ovary Syndrome and Quality of Life scale and subscale scores regarding symptoms (n=249).

	Hair growth		Menstrual irregularity		Obesity		Acne-pimples		Hair loss		Insulin resistance		Hormone imbalance		Fatigue	
	Mean	SD	Mean	SD	Mean	SD	Mean	SD	Mean	SD	Mean	SD	Mean	SD	Mean	SD
Psychosocial and emotional	3.22	0.79	3.10	0.84	3.27	0.80	3.31	0.78	3.20	0.85	3.27	0.87	3.30	0.80	3.15	0.84
Fertility	2.55	1.04	2.55	1.02	2.54	0.99	2.61	1.08	2.54	1.10	2.62	1.07	2.71	1.11	2.50	1.06
Sexual function	2.43	0.88	2.35	0.93	2.40	0.95	2.48	0.96	2.42	0.91	2.51	1.01	2.56	0.95	2.36	0.91
Obesity and menstrual disorders	3.34	0.96	3.30	0.96	3.63	0.81	3.34	0.99	3.33	1.01	3.59	0.88	3.45	0.99	3.30	0.98
Hair growth	3.85	1.02	3.25	1.27	3.42	1.36	3.51	1.28	3.51	1.27	3.42	1.34	3.54	1.30	3.32	1.36
Coping	2.84	0.99	2.66	1.01	2.84	1.00	2.87	1.04	2.80	1.05	2.91	1.04	2.87	1.05	2.72	1.03
PCOSQ total	3.08	0.71	2.92	0.76	3.08	0.73	3.08	0.76	3.02	0.79	3.11	0.79	3.12	0.76	2.95	0.79

The logistic regression model established to examine the effect of variables on PCOSQ total was found significant (p<0.05). When the results were examined, age had a negative effect on PCOSQ total (β=-0.446, p<0.05), whereas frequency of going to the hospital for PCOS control had a positive effect (β=0.163, p<0.05). Age and frequency of going to the hospital for PCOS control explained 18.6% of the change in PCOSQ total (Table 3).

**Table 3:** Evaluation of variables affecting women's Polycystic Ovary Syndrome and Quality of Life total score by logistic regression.

Dependent variable	Independent variable	Non-standardized		Standardized	t	p	R^2^
		B	SE	Beta			
PCOSQ total	Fixed	3.589	0.883		4.063	0.000*	0.186
	Age	-0.337	0.066	-0.446	-5.137	0.000*	
	Education status	-0.135	0.144	-0.066	-0.942	0.348	
	Marital status	0.214	0.122	0.139	1.752	0.081	
	Employment status	0.087	0.110	0.055	0.789	0.431	
	Income status	0.081	0.081	0.067	1.009	0.314	
	Currently having any disease other than PCOS	-0.194	0.109	-0.119	-1.782	0.076	
	Diagnosis of PCOS (years)	0.129	0.069	0.134	1.871	0.063	
	Someone in the family diagnosed with PCOS	-0.101	0.121	-0.055	-0.832	0.406	
	Frequency of hospitalization for PCOS controlled	0.163	0.067	0.163	2.447	0.015*	
	Use of nutritional supplements	-0.143	0.116	-0.081	-1.237	0.218	

Model: f=4.622, p=0.000.

* p>0.001.

## DISCUSSION

This study showed that the most important symptoms affecting the quality of life of women with PCOS were menstrual irregularity and fatigue, while the least affected areas were hormone irregularity and insulin resistance. At the same time, in this study, it was observed that women were irregular and overfed and neglected their controls. In women, the age variable was found to affect all sub-dimensions of the PCOSQ scale except sexuality. Women aged 26–30 years had higher PCOSQ total scores, and regression analysis showed that the total scores were negatively affected by increasing age.

It is difficult to confirm the diagnosis of PCOS, as it is associated with metabolic dysfunction and is a risk factor for the development of type 2 diabetes cardiovascular diseases and endometrial cancer. Therefore, for diagnosis, according to many studies, anovulation and hyperandrogenism are required^14,15^. In addition, it is necessary to evaluate the clinical, hormonal, and metabolic features to understand the impact of different phenotypes on PCOS^16,17^. There is considerable evidence that women with PCOS have more regular menstrual cycles, decreased serum androgen levels, and improved polycystic ovarian morphology with increasing age^18^. However, another study stated that the decrease in androgen levels over time did not show metabolic improvement and was a risk factor for type 2 diabetes according to phenotypes^16^. A study of women with PCOS between the ages of 31 and 46 years emphasized that these women had significantly lower life quality^19^. In this study, women over 36 years had the lowest life quality scores. This may be due to increased fat accumulation over the years, negative body image, and metabolic problems. When developing treatments to improve quality of life, it is important to understand the factors that reduce quality of life in women with PCOS^20^. Long-term treatments and evaluation of hormonal and metabolic parameters of these women are important for their quality of life^21^.

Menstrual irregularity is a common gynecologic condition affecting women with PCOS, especially in early reproductive age. A meta-analysis of studies with women with PCOS emphasized that hirsutism and menstrual imbalance affect women's quality of life. Up to 90% of women with PCOS report facial hair as a problem. Women with PCOS who experience hirsutism report feeling “unfeminine,” “weird,” “strange,” “weird,” and “different”^18^. As a smooth, hairless body or face and regular menstruation are associated with femininity and fertility, women with hair growth/menstrual irregularity symptoms feel “different” and less “feminine” than other women^22,23^. Young women may feel less sexually attractive because of their appearance. Negative affect may be accompanied by anxiety and depression. Women under 25 and over 30 years had a lower quality of life due to menstrual irregularities. Women's long and painful menstrual cycles may have affected their quality of life, and even if they used medication, the side effects may have caused discomfort for them. In addition, childlessness is an important problem in Turkish society. Women's perception that menstrual irregularity would jeopardize their fertility in later life may have caused them anxiety.

Sleep disturbances and fatigue are very common in women with PCOS in conditions of inadequate physical activity. In a recent study, women with PCOS reported sleep difficulties and occasional restless sleep. In the same study, women often reported severe fatigue^23^. In another study, it was reported that 29.5% of women with PCOS complained of sleep disorders, and 64% had been struggling with this problem for at least 1 year^20^. Therefore, optimizing sleep may be important to support and maintain healthy lifestyle changes for women with PCOS. In this study, women who reported being tired had a lower quality of life. However, the association between PCOS and diet quality may only be useful if women are able to get enough quality sleep. Overnutrition closure increases with decreased sleep duration. It is, therefore, important to normalize sleep duration through diet and exercise to focus on quality lifestyle behaviors. Insulin resistance may be exacerbated by sleep disturbances in women with PCOS and their energy balance may be disturbed.

## CONCLUSION

In women with PCOS, quality of life decreases with age. Menstrual irregularity and fatigue affect women the most. These women eat irregular diets and neglect their check-ups. We recommend utilizing the available evidence and developing appropriate action plans according to age.

### Limitations of the study

The diagnosis of menstrual irregularity was based on a self-reported questionnaire, so there may be a risk of information bias in reporting symptoms. Moreover, there was no ovarian ultrasonography to help diagnose PCOS and no clinical assessment of hyperandrogenism.
